# Thermal and Electrical Conduction of Single-crystal Bi_2_Te_3_ Nanostructures grown using a one step process

**DOI:** 10.1038/srep19132

**Published:** 2016-01-11

**Authors:** Dambi Park, Sungjin Park, Kwangsik Jeong, Hong-Sik Jeong, Jea Yong Song, Mann–Ho Cho

**Affiliations:** 1 Institute of Physics and Applied Physics, Yonsei University, Seoul, 120-749 Korea; 2 School of Integrated technology, Yonsei University, Incheon, 406-840 Korea; 3 Korea Research Institute of Standards and Science, Daejeon 305-340.

## Abstract

Single-crystal Bi_2_Te_3_ nanowires (NWs) and nanoribbons (NRs) were synthesized by a vapor-liquid-solid (VLS) method from Bi_2_Te_3_ powder. To investigate the thermal properties of the Bi_2_Te_3_ nanostructure, a nondestructive technique based on temperature dependent Raman mapping was carried out. The Raman peaks were red shifted with increasing temperature. In addition, the fraction of the laser power absorbed inside the Bi_2_Te_3_ nanostructures was estimated by optical simulation and used to calculate the thermal conductivity value (κ). The thermal conductivity value obtained for the Bi_2_Te_3_ NW and NR was 1.47 Wm^−1^K^−1^ and 1.81 Wm^−1^K^−1^ at 300 K, respectively. The electrical conductivity of the Bi_2_Te_3_ nanostructure was also measured. In particular, an excellent electrical conductivity value of 1.22 * 10^3 ^Ω^−1^ *cm*^−1^ was obtained for the Bi_2_Te_3_ NW at 300 K. This result can be attributed to topological insulator surface states. As a result of our study, the figure of merit (ZT) for the Bi_2_Te_3_ NW and NR can be significantly improved.

Thermoelectric (TE) devices have recently attracted a great deal of interest because of their potential applications in energy harvesting, chip cooling and sensing. Thermoelectric (TE) power generation devices play an important role in utilizing solar and geothermal power more efficiently, as well as in capturing automobile-derived heat via a noise-free and low-maintenance conversion process^1^,^2^,^3^. In order to use TE devices in such applications, attaining a high efficiency is the most important issue. Therefore, many studies have been made in attempts to obtain a high efficiency through the development of new materials and device modification^4^,^5^,^6^,^7^.

Thermoelectric performance is typically evaluated by a dimensionless figure of merit (ZT), defined as ZT = S^2^σT/κ, where S is the Seebeck coefficient, σ is electrical conductivity, T is absolute temperature, and κ is thermal conductivity^8^. To improve the figure of merit, the three factors, namely the simultaneous increase in electrical conductivity, increasing thermoelectric power, and decreasing thermal conductivity need to be controlled. In order to achieve a conversion efficiency by replacing the general cooling-generation technology a ZT of 4 is needed. However, at present, ZT = 2 represents the practical limit for conventional structure and materials. Therefore, recent studies have focused on reducing the dimensionality of typical materials into the nanoscale range because both a higher density of states and an increased phonon scattering or reduced lattice thermal conductivity can be achieved in low-dimensional structures^9^. Bi_2_Te_3_ is a well-known material that can be used as a semiconductor, given its low lattice thermal conductivity and, simultaneously, a quite high electrical conductivity. Therefore, it has been actively studied in attempts to increase the ZT value through reducing the dimensions into a nano structure.

In the measurement of thermal conductivity in nanostructures, the 3-omega method is generally used. However, the sample is destroyed during the measurement, which makes device fabrication difficult^10^,^11^,^12^. Moreover, thermal contact resistance is a critical problem in terms of extracting the intrinsic thermal conductivity using nearly all of the steady-state heat flow methods. Therefore, noncontact approaches using optical spectroscopy have been introduced to measure the thermal conductivity of individual semiconductor nanowires (NW)^13^,^14^,^15^, nanotubes^16^, any layered structures such as MoS_2_^17^ and graphene^18^. This method is based on the heat flow caused by the laser heating of freely suspended nanostructures for the determination of the local temperature by micro-Raman spectroscopy^13^,^14^,^15^,^16^,^17^,^18^.

Recently, as a typical one-dimensional (1D) nanostructure material, Bismuth telluride (Bi_2_Te_3_) NWs and nanoribbon (NR) that have potential applications in electronic, thermoelectric, and topological insulator devices have gained the spotlight^19^. In particular, Bi_2_Te_3_ based alloys are known to be excellent thermoelectric materials at room temperature^20^. The most frequently used method for synthesizing Bi_2_Te_3_ is template-assisted electrodeposition. However, such solution-based chemical methods intrinsically have a contamination issue^21^,^22^. On the other hand, high quality bismuth telluride nanostructures can be synthesized using the vapor-liquid-solid (VLS) method. Although the growth of bismuth telluride nanorods and nanobelts by the VLS method has been reported by Wang *et al.*^23^ and Wei *et al.*^24^, single-crystalline nano structures prepared using the VLS method have not yet been reported^23^. The single crystalline growth of bismuth telluride NWs by the VLS method using an additional post-annealing process in a Te atmosphere has been reported by Bacel Hamdou^25^. Unfortunately, the synthesis of a single-crystalline Bi_2_Te_3_ NW using the VLS method without an additional annealing process has not been reported yet. In this work, we attempted to determine the growth window needed for preparing single-crystalline NW using VLS. We carried out the one-step epitaxial growth of bismuth telluride nanostructures using a Au catalytic VLS method. Finally, we were able to determine the growth window needed for preparing Bi_2_Te_3_ nano structures with the shape of NWs and NRs within very narrow process temperature parameters without the need for any additional processing^26^. In addition, we successfully measured the thermal conductivity к of a, individual single crystal Bi_2_Te_3_ NW and NR using an optical method based on laser heating. To apply the optical method to the measurement for the thermal properties of Bi_2_Te_3_ nanostructures, Raman spectra of Bi_2_Te_3_ nanostructures at various temperatures were collected. Using the temperature-dependent Raman spectra, we were able to successfully extract the thermal conduction parameters. The obtained thermal conductivity value of Bi_2_Te_3_ NW and NR was 1.47 Wm^−1^K^−1^ and 1.81 Wm^−1^K^−1^ at 300 K, respectively. Additionally, we determined that the average electrical conductivity value of Bi_2_Te_3_ NW and NR is increased up to 1.22 * 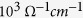 and as 0.67 * 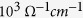 at 300 K, respectively. Although the thermal conductivity values of the Bi_2_Te_3_ nanostructures were very similar to previously reported values, the electrical conductivity values of the Bi_2_Te_3_ nanostructures were significantly increased, compared to the reported values. These results indicate that the control of electrical conductivity rather than thermal conductivity needs to be carefully considered in the design of various devices such as nanostructures based electronics, optics, sensor, and thermoelectronics in which Bi_2_Te_3_ nano structures are used^27^.

## Results and Discussion

**Figure 1:**
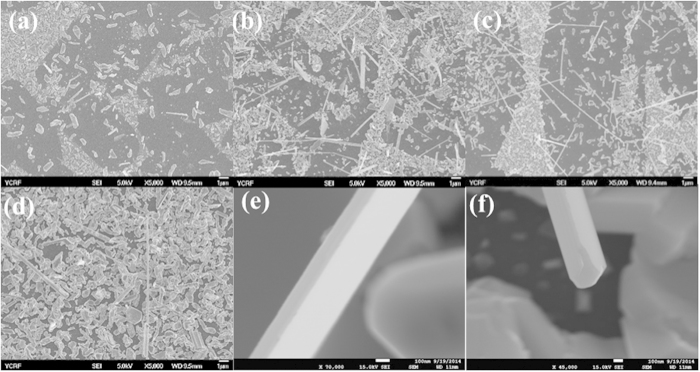
SEM images of as-grown Bi_2_Te_3_ nanostructures as growth temperature of (**a**) 275 °C, (**b**) 280 °C, (**c**) 285 °C and (**d**) 290 °C. SEM images (**e**) of Bi_2_Te_3_ NR and (**f**) of Bi_2_Te_3_ NW synthesized by a VLS method using Au as a catalyst at a gas flow rate of 50:10 sccm (Ar : H_2_).

Figure 1(a)~(d) show SEM images of as-grown Bi_2_Te_3_ nanostructures for growth temperatures ranging from 275 to 290 °C with a temperature increment of 5.0 °C. In the growth temperature region from 275 to 290 °C, clusters of the Bi-Te alloy were commonly observed as shown, in Fig. 1(a)~(d). At a relatively low growth temperature of 275 °C, the cluster corresponding to a pre-step for nanostructure growth is generated. However, at the relatively high growth temperature of 290 °C, clusters which are close to the film shape are generated, with a few nanostructures being observed. Finally, the nano structure was grown within the very narrow temperature window between 280 °C and 285 °C. High-magnitude images in Fig. 1(e,f) show that the shape of the nanostructure is different and can be distinguished between the growth windows of 280 °C and 285 °C: i.e., Bi_2_Te_3_ NRs and NWs were grown only at 280 and 285 °C, respectively. An examination of the TEM showed that the synthesized NW, has a square shape, and that the diameters d of Bi_2_Te_3_ nanowires are distributed between 50 nm and 90 nm (supporting Fig. 2a). The width and thickness of the Bi_2_Te_3_ NR which has rectangular shape is ~200 nm and ~80 nm, respectively (supporting Fig. 2b). The length of Bi_2_Te_3_ NW and NR is range from 4 μm to 13 μm (supporting Fig. 2c). These results indicate that the growth condition used to prepare a Bi_2_Te_3_ NW and NRs are very sensitive to minor temperature changes. Although the one-step growth of Bi_2_Te_3_ NWs and NRs with a single crystalline structure has not been easily accomplished, we have successfully grown Bi_2_Te_3_ nanostructures within a very narrow temperature range.

**Figure 2:**
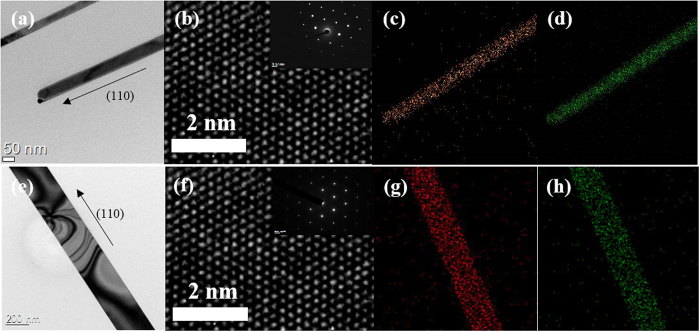
TEM images (**a**) and (**b**) of Bi_2_Te_3_ NWs and (**e**) and (**f**) of Bi_2_Te_3_ NRs show a defect-free crystal structure. Inset of (**b,f**) shows the corresponding SAED pattern of the Bi_2_Te_3_ NWs and NRs with axis along [110]. EDX results of (**c,d**) the Bi_2_Te_3_ NWs and (**g,h**) Bi_2_Te_3_ NRs grown under different growth temperature. Bi, Te elements are uniformly distributed over the whole nanostructures. In the EDX results, the red shows Bi and the green shows Te.

In order to investigate the crystalline structure and stoichiometry of the grown nano structures, we collected TEM and EDX data, respectively, as shown in Fig. 2 (a–h). The 2:3 ratio of Bi and Te atoms in the NWs and NRs was confirmed by EDX. TEM analyses show that the Bi_2_Te_3_ nanostructures exhibit a rhombohedral crystal structure represented by a distorted structure of the simple cubic crystal. All of the Bi_2_Te_3_ nanostructures were grown as single crystalline materials with a growth direction of [011]. HR-TEM images show clear lattice fringes from a 0.22 nm spacing on the (011) lattice planes of the rhombohedral Bi_2_Te_3_ structure, which is consistent with previously reported findings for Bi_2_Te_3_ nanostructures.

**Figure 3:**
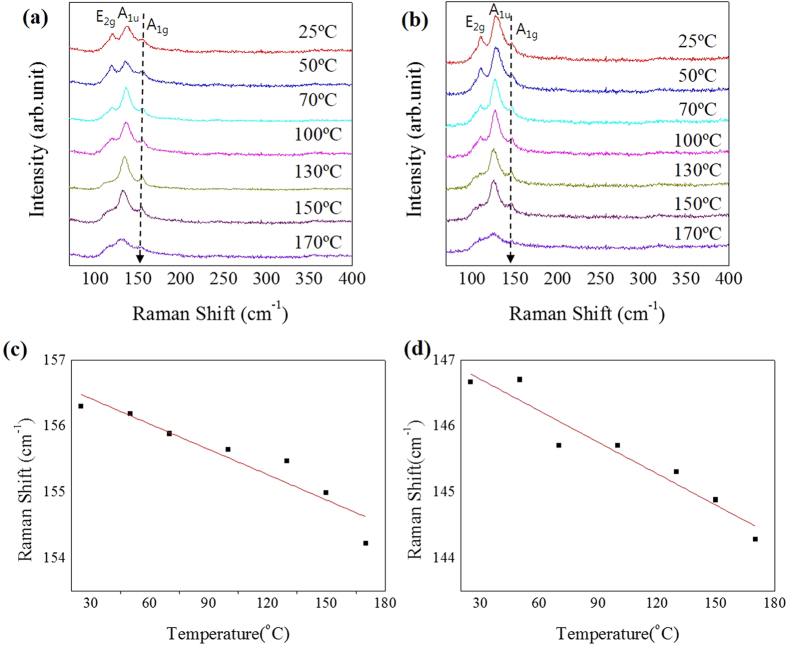
Temperature-dependent Raman spectra for Bi_2_Te_3_ (a) NWs and (**b**) NRs. Raman frequencies of A_1g_ modes in Bi_2_Te_3_ (**c**) nanowire and (**d**) nanoribbon as temperature increased.

To investigate the thermal properties of the Bi_2_Te_3_ nanostructures, we collected Raman spectra at various temperatures using Linkam stages which permit the temperature to be controlled. Figure 3(a,b) show Raman spectra of the Bi_2_Te_3_ NW and NR, respectively, increasing temperature from 25 to 170 °C. As seen in the Fig. 3(a,b), three prominent peaks are observed, which correspond to E_2g_, A_1u_ and A_1g_ modes. All of the Raman active modes in the bulk structure also appear in the nano structure. However, some of the Raman active modes in the nano structure are not observed in the bulk structure because of the reduction or the breaking of the symmetry in the nanowire: i.e., the A_1u_ mode for Bi_2_Te_3_ shows up only in low dimensional structures as the result of the breaking of the symmetry of Bi_2_Te_3_^28^. That is, typical Raman activity modes controlled by selection rules reflect the difference in the structural symmetry between the nano structure and the bulk. In addition, a blue shift occurred in the Raman frequency as the dimension decreased, because the vibrational properties change with a decrease in the dimensions of the materials^28^. That is, a volume contraction occurs in the nanostructure due to the size-induced radial pressure, which leads to an increase in the force constants. In vibrational transitions, the wavenumber varies approximately in proportion to the force constant (k)^1/2^. Consequently, the Raman shift towards a higher wavenumber due to the increasing force constants. For this reason, the Raman peaks of Bi_2_Te_3_ NWs are observed at higher frequencies than those of Bi_2_Te_3_ NRs in this study. As the temperature increases, because Raman peak position and full width at half maximum (FWHM) are governed by phonon anharmonicity, all of Raman modes soften linearly and clearly broaden. In the nanostructures, the change in the Raman peak position can be more reliably detectable than that of FWHM, because the FWHM is very sensitive to changes in beam focus and the position of the laser spot. Therefore, in this study, we considered only temperature dependence of the peak position. In addition, because the direction of heat transfer in the nanostructures is in the growth direction, we especially focused on the A_1g_ peak shift.

Figure 3(c) shows a plot of Raman peak shift versus temperature for the Bi_2_Te_3_ NW. As the temperature increases, the change in the A_1g_ peak clearly shows a linear shift and becomes broadened. In the case of Bi_2_Te_3_ NW, the A_1g_ peak that appeared at 156.3 cm^−1^ is shifted to 154.2 cm^−1^ with a temperature change from 25 °C to 170 °C. As a result, the peak is shifted in proportion to the temperature and the slope of the fitting line is the temperature dependence coefficient, as shown in Fig. 3(c). The measured slopes from the linear fitting of the phonon shift for Bi_2_Te_3_ NWs and NRs are almost the same: i.e., the fitting values are −0.01281 ± 0.00197 cm^−1^/^o^C for NWs and −0.0158 ± 0.00106 cm^−1^/^o^C for NRs. The change in phonon frequency with temperature results from the anharmonic terms in the lattice potential energy determined by the anharmonic potential constants, the phonon occupation number, and the thermal expansion of the crystal^29^. Since the anharmonic term in Lagrangian for the lattice vibrating could cause the Raman shift to have a first-order (T) and second-order (T^2^) relation with temperature, the temperature dependence of Raman frequency is given by


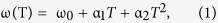


where 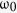 is the harmonic frequency of the Raman mode, 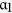 and 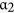 are the first- and second-order temperature coefficients, respectively. The temperature dependence of the Raman shift can be written as a result of two additional effects: one being the “self-energy” shift caused by the anharmonic coupling of the phonon modes and the other is the thermal expansion of the crystal. Therefore, the Raman shift is given by


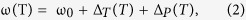


where 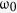 is the harmonic frequency of the optical mode, 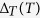 is the Raman frequency shift caused by thermal expansion, 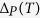 is the Raman frequency shift caused by anharmonic phonon-phonon coupling. 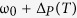 is equal to the frequency of Raman mode at T = 0K. The observed linear relationship for a Raman peak shift as a function of temperature in the Bi_2_Te_3_ NW and NR is caused by the anharmonic vibrations of the lattice, which mainly includes contributions from the thermal expansion of the lattice due to the anharmonicity of the interatomic potential^30^. As the lattice expands with increasing temperature, the equilibrium positions of the atoms and the interatomic forces change. As a result, a shift in phonon energy occurs. At temperatures above room temperature, since thermal expansion mainly influences the Raman peak shift, the relationship between the Raman peak shift with increasing temperature is typically linear. Therefore, the equation for a temperature-dependent Raman peak shift can be expressed by ∆ω = α∆T, where α, the slope of the temperature dependence, is the first-order temperature coefficient for the Raman mode, and T is the temperature. That is, a temperature-dependent Raman study as the noncontact method to monitor the local temperature can provide information on the thermal conduction of the nanostructure.

**Figure 4:**
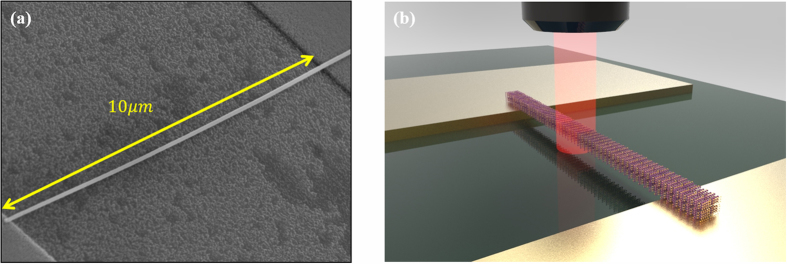
SEM image suspended (**a**) Bi_2_Te_3_ NW on the two pads. (**b**) Sketch of suspended Bi_2_Te_3_ nanostructure on Ti line array with 10 μm width and 150 nm height.

**Figure 5:**
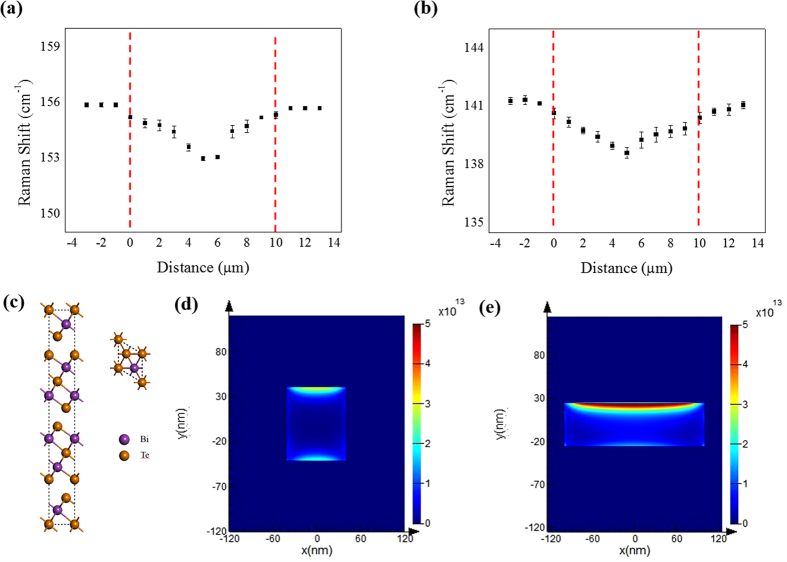
Raman measurement on a single (**a**) Bi_2_Te_3_ NW and (**b**) NR. Example of parabolic A_1g_ mode profiles obtained for homogeneous nanostructures. Symbols indicate experimental data points. (**c**) The rhombohedral conventional unit cell of Bi_2_Te_3_. 2-D FDTD simulation results of the Bi_2_Te_3_ (**d**) NW and (**e**) NR for no substrate configurations, respectively. The estimated value of laser power absorbed in NW and NR is 1.792 * 10^−7^ *W*and 1.8186 * 10^−6^ *W*, respectively.

To separate substrate effects from the Raman characteristics, we measured the Raman A_1g_ mode of suspended Bi_2_Te_3_ nanostructures. Figure 4(a) shows Bi_2_Te_3_ NW suspended on linear type electrodes. A schematic diagram of Raman measurement is shown in Fig. 4(b). Since there is a thermal heat sink when the nanostructure is suspended in Ti metal, a downshift in the Raman spectra is observed in the center of the suspended nanostructure, compared to the corresponding value for the ends. The heat in the nanostructure generated by a laser flows into both directions in the metal pads. In Fig. 5(a), an example of Raman spectra obtained during a scan along a NW length direction clearly shows a position-dependent shift in the A_1g_ peak. Using the A_1g_ peak shift between two points from the near pad to the mid-point of the suspended NW, maximum temperature difference (ΔT_max_) of 305.65 K could be obtained in the NW. In the case of NR, the position-dependent shift of the A_1g_ peak could be also measured using a method to that used in the case of the NW, as shown in Fig. 5 (b). Finally, ΔT_max_ of 220.54 K could be calculated in NR.

We used a simple model to describe the temperature profile on the suspended part of the nanostructure that was developed by Hsu *et al.*^31^


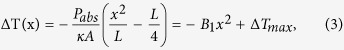


with 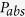 the power absorbed inside the nanostructure, L the length of the suspended part, A the cross section area, and B_1_ the curvature. Finally, we used 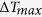 values to obtain the thermal conductivity 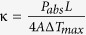.

Where the 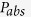
is estimated using the optical constant n (refractive index) and k (extinction coefficient) for Bi_2_Te_3_ at 633 nm, obtained through FDTD simulations. The n (6.96) and k (5.61) values in a rhombohedral Bi_2_Te_3_ single crystal were obtained using the DFT method. (Supporting Fig.3) Using the morphological data for Bi_2_Te_3_ NW and NR measured by AFM (Supporting Fig.2), the absorption of the laser light by the nanostructure could be obtained, as shown in Fig. 5(c,d) for the 2-D FDTD simulation results of Bi_2_Te_3_ NW and NR, respectively. As a result, the powers absorbed inside NW and NR are 6.45281 * 10^−7^ *W* and 1.8186 * 10^−6^ *W*, respectively.

Using these values, thermal conductivity of Bi_2_Te_3_ NW and NR at room temperature could be obtained: i.e., 1.47 W/mK for NW and 1.81 W/mK for NR (minimum thermal conductivity of the measured data; 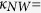
1.43 W/mK, 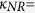
1.79 W/mK). These thermal conductivity values are similar to data obtained using the 3-omega method^32^. Moreover, comparing the two values for the NW and NR samples, the dependence of morphology on thermal conductivity can be also observed: i.e., the thermal conductivity value of Bi_2_Te_3_ NW is less than the value of NR. Simply, concerning the morphological characteristics, the difference is caused by the surface-to-volume ratio because the surface-to-volume ratio of NR is smaller than that of NW. In a nanostructure, since the phonon scattering process mainly occurs at the surfaces and interfaces, the thermal conductivity generally decreases with increasing surface-to-volume ratio^8^,^33^.

**Figure 6:**
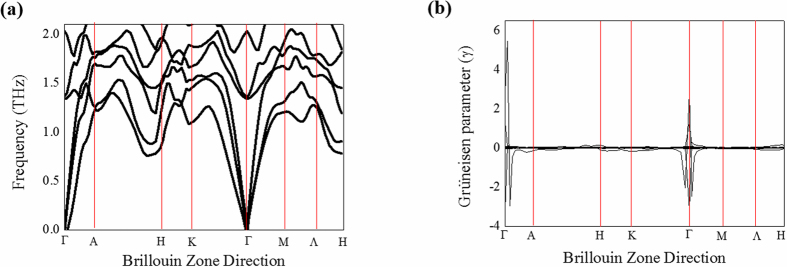
Theoretically calculated phonon and Grüneisen dispersions. (**a**) Phonon dispersion in the acoustic mode. (**b**) The acoustic mode Grüneisen parameters. The Grüneisen parameter at the Γ-Α Brillouin zone direction is very high. High Grüneisen parameter value indicates the strong anharmonicity and this leads to low thermal conductivity.

In order to investigate the difference in thermal conductivity between NR and NW in detail, we checked the anharmonicity in NW and NR samples with a highly ordered crystalline structure. It is known that strong anharmonicity in bonding can be generated, which decreases lattice thermal conductivity in ordered crystal structures. In order to find the difference in the lattice anharmonicity for the estimation of thermal conductivity between Bi_2_Te_3_ NW and NR, we calculated phonon dispersions and Grüneisen parameters using first-principles density functional theory (DFT) phonon calculations within a quasi-harmonic approximation. Phonon dispersion was calculated with a hexagonal 2 × 2 × 1 supercell the geometry of which was an optimized Bi_2_Te_3_ unit cell. For the phonon calculation, we used a 500 eV cut-off energy and a 5 × 5 × 1 k-point. All k-point spacings were chosen to be less than 0.25 1/Ang. Figure 6(a) shows the calculated phonon dispersion in Bi_2_Te_3_. We calculated the Grüneisen parameters from the definition γ = −dlnω/dlnV, where ω and V are the phonon frequency and the crystal volume, respectively. To obtain the acoustic mode Grüneisen parameter, we used additional phonon frequencies for two different crystal volumes: a crystal volume at equilibrium and a volume that was increased by 1%. The Grüneisen parameters at the Γ-K and Γ-H Brillouin zone direction are considerably lower than the Γ-Α Brillouin zone direction, as shown in Fig. 6(b). Consequently, the NWs are mostly influenced by the Γ-Α Brillouin zone direction in the z-axis, while the NRs are not influenced by the Γ-Α Brillouin zone direction but, rather, by the Γ-K and Γ-H Brillouin zone direction. The soft mode of the Γ-Α Brillouin zone direction along the z-axis suggests weak interatomic bonding and possible strong anharmonicity. A high Grüneisen parameter value indicates strong anharmonicity, resulting in a low thermal conductivity. For this reason, the thermal conductivity value of Bi_2_Te_3_ NW is lower than that of NR. That is, the Grüneisen parameters well characterize the relationship between phonon frequency and changes in crystal volume.

**Figure 7:**
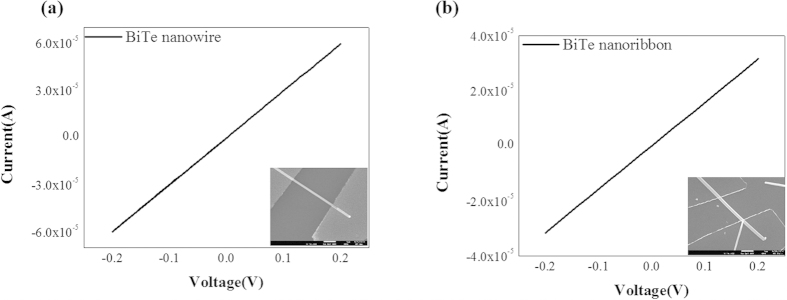
I-V characteristics for a (**a**) Bi_2_Te_3_ NW and (**b**) NR. The resistance of NW 3.13 k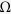 and NR is 1.82 k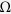. The electrical conductivity of NW 1.525 * 10^3^ Ω^−1^ *cm*^−1^ and NR is 0.86 * 10^3^ Ω^−1^ *cm*^−1^. (**c**) The electrical conductivity of Bi_2_Te_3_ NW and NR. The value of Bi_2_Te_3_ NW is higher than NR over the double. (**d**) The resistivity of Bi_2_Te_3_ NW and NR. The resistivity of the Bi_2_Te_3_ single crystal NW is significantly decreased to 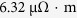. The resistivity of NW was lower than the NR because of a high surface to volume ratio.

Figure 7 shows the electrical properties of Bi_2_Te_3_ single crystal nanostructures measured in room temperature. The electrical contacts between the Bi_2_Te_3_ nanostructure and electrodes are ohmic. Using the measured resistance values for NW and NR, a significantly increased electrical conductivity of the our Bi_2_Te_3_ single crystal NW, 1.525 * 10^3^ Ω^−1^ *cm*^−1^, was obtained at room temperature (average electrical conductivity of the measured data 1.22 * 10^3^ Ω^−1^ *cm*^−1^), which is a considerably higher than that of the on-film formation of nanowires (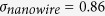 * 10^3^ Ω^−1^ *cm*^−1^)^34^, bulk material (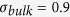 * 10^3^ Ω^−1^ *cm*^−1^)^35^,^36^ and the thin film (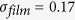 * 10^3^ Ω^−1^ *cm*^−1^)^37^. The electrical conductivity of our Bi_2_Te_3_ single crystal NR was also increased to 0.86 * 10^3^ Ω^−1^ *cm*^−1^at room temperature (average electrical conductivity of the measured data = 0.67 * 10^3^ Ω^−1^ *cm*^−1^), which is lower than that of our NW but notably higher than that of the thin film. The difference in electrical conductivity between NW and NR is the result of a dimensional effect: i.e., the dimension of NW is less than that of NR. Considering the difference in the cross-sectional area between NW and NR, the difference is not consistent with the difference in electrical conductivity. Comparing the crystalline structure with the reported, the above experimental results showing a high electrical conductivity are caused by the highly ordered crystalline structure of the Bi_2_Te_3_ NWs and NRs grown using VLS method. The highly ordered structure can generate a specific surface effect^38^. At surfaces crystalline solids, the physical properties differ from the bulk ones. The physical properties of crystalline surfaces are different from those for bulk surfaces. Due to the lack of bonding neighbor atoms at the surface, the degeneration of the energy at the surface is different from that for a bulk structure. Naturally, from the surface band structure, a surface density of states is induced. Depending on the relative position of the surface states with respect to the bulk band structure, charges need to be transferred from the bulk to the surface states or vice versa^39^,^40^,^41^. For a well-known narrow band gap semiconductor such as Bi_2_Te_3_ (~0.15 eV), there are surface states at an energetic position above the conduction band edge, which leads to a charge transfer from the surface state into the bulk^41^,^42^. Thus, an electron layer accumulates at the surface region, leading to significantly increased electrical conductivity of the Bi_2_Te_3_ nanowire. In particular, the surface state of the NW is more enhanced than that of NR because the NW has a higher surface-to-volume ratio than that of the NR. In our study, the resistivity of the Bi_2_Te_3_ single crystal NW was found to be significantly decreased to 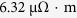. The repeated and consistent results indicate that the measured value reflects the intrinsic characteristics of the well-ordered single crystalline NW. The different behavior of NW in terms of thermal and electrical conduction strongly suggests that Bi_2_Te_3_ NW promises to be a most efficient structure for use in thermoelectric devices.

## Conclusions

In conclusion, we report herein on the one step VLS growth of Bi_2_Te_3_ nanostructures and the calculation of its thermal conductivity using Raman spectroscopy for the first time. Through this growth method, single-crystal, defect-free, and rhombohedral crystal structure Bi_2_Te_3_ NWs and NRs were synthesized. Using temperature-dependent A_1g_ peak shifts in Raman spectra of suspended Bi_2_Te_3_ nanostructures, the thermal conductivity could be successfully determined. Moreover, we demonstrated the power absorbed in the single crystal Bi_2_Te_3_ nanostructures using FDTD simulation. From these results, the evaluated thermal conductivity of Bi_2_Te_3_ NW and NR is 1.47 W/mK and 1.81 W/mK at room temperature, respectively. Through the change in Grüneisen parameters with the surface-to-volume ratio, we were able to determine that the difference in thermal conductivity between NW and NR is caused by the strong anharmonicity. That is, since the scattering of phonons is significantly increased at surfaces and interfaces, the anharmonicity in NW is enhanced, compared to that in NR. Therefore, the thermal conductivity of Bi_2_Te_3_ NWs is lower than Bi_2_Te_3_ NRs. In particular, in a well-ordered single crystalline NW, the electrical conductivity is greatly enhanced, while the thermal conductivity obtained by an optical method is close to the reported value measured by a three-omega method. The measured resistivity value indicates that electrical conduction mainly occurs on the surface, not in the bulk. This result is related to the surface charge density of nanowires. Finally, based on our experimental results, a high thermoelectric figure of merit can be obtained in the case of single crystal Bi_2_Te_3_ nanostructures. The accumulated surface charges can be critical in terms of enhancing the electrical conductivity of a system, suggesting the possibility for their applications in thermoelectric devices.

## Methods

### Experiment

Bi_2_Te_3_ NWs and NRs were synthesized by the VLS method in a 12 in. horizontal tube furnace equipped with a 1 in. diameter quartz tube. Based on the melting temperatures of Bi_2_Te_3_ powder and the temperature profile of the tube furnace, the location of the precursors was adjusted appropriately to optimize the synthesis of the Bi_2_Te_3_ nanostructures. Bi_2_Te_3_ powder was placed at the center of the upstream zone (570 °C). A 3-nm thick Au film (as a catalyst) was deposited on a cleaned Si (001) substrate in a metal growth chamber at a growth pressure of ~3 × 10^−7^ torr by thermal evaporation. A Si substrate covered with Au films was placed in the downstream zone of the furnace. Before we raise the temperature, the quartz tube was evacuated to below 2.4 Torr and flushed repeatedly with Ar/H_2_ (50/10 sccm) gas to minimize oxygen contamination. For the growth of Bi_2_Te_3_ NW and NR, we controlled the temperature of the downstream zone in furnace from 275 to 290 °C. We found the optimized temperature conditions for preparing NWs and NRs to be: 285 °C for NWs and 280 °C for NRs. The heating rate was controlled to 20 °C/min and the growth time for the nano structures was set to 10 hr. The morphological characteristics and crystalline structures of the Bi_2_Te_3_ NWs and NRs were analyzed by scanning electron microscopy (SEM) and high-resolution transmission electron microscopy (HR-TEM). Energy dispersive X-ray (EDX) analysis verified the chemical composition of the Bi_2_Te_3_ nanostructures. To investigate the thermal properties of the Bi_2_Te_3_ nanostructures, temperature dependence of Raman spectra of the nanostructures were obtained by Raman spectroscopy (Nanofinder 30, Tokyo instrument). Raman spectra at various temperatures were measured using Linkam stages which permit the temperature to be controlled. The applied laser light had a wavelength of 633 nm and the acquisition time of each Raman spectrum was 20 s. In order to determine the optimized laser conditions, we investigated the Raman characteristics for structural stability with irradiated laser power. The optimized laser power was 0.07 mW and the diameter of the laser beam was ~1 μm, which is a very stable condition in which there is no structural deformation in the Raman spectra (supporting Fig. 1). The NWs and NRs were mechanically transferred from a silicon substrate containing large arrays of lined Ti pads of 10 μm width. The pads were 150 nm high and were fabricated by e-beam evaporation after a standard photolithography step. For the measurements, we selected NWs and NRs which are freely suspended between two pads at a length of 11~13 μm. The samples were positioned on a piezo stage to scan along the NW and NR with a step size of 1 μm. The polarization of the excitation light was set parallel to the NW and NR axis in order to maximize absorption and thus heating effects as well as the Raman scattering intensity. The power absorbed inside the nanostructure was estimated using the optical constant n (refractive index) and k (extinction coefficient) for Bi_2_Te_3_ at 633 nm, obtained by FDTD calculations. To find the values for n and k for Bi_2_Te_3_, we used first-principles density functional theory (DFT) phonon calculations within the quasi-harmonic approximation. We performed ab initio calculations using the Vienna ab initio simulation package (VASP)^26^ with the generalized gradient approximation (GGA) using the PBEsol approximation^27^. MEDEA Software was used as GUI. In all calculations, Projected Augmented Wave (PAW) peudopotentials were employed with a plane wave basis set with a cutoff of 500 eV and a convergence of 1E^−6^ eV. In all geometric estimations, the forces on each atom were relaxed to less than 0.02 eV/Å. The initial unit cell of Bi_2_Te_3_ rhombohedral cell was geometrically optimized with a 7 × 7 × 7 k-point. Phonon dispersion calculated with a hexagonal 2 × 2 × 1 supercell of geometry optimized the Bi_2_Te_3_ unit cell. The lattice parameters (a and c for the Bi_2_Te_3_) obtained here are in reasonable agreement with available experimental data. To study the electrical properties of the Bi_2_Te_3_ nanostructures, the nanostructures were transferred by a dropcasting method to a p-type Si substrate with a 50 nm SiO_2_ insulating surface layer. Scanning electron microscopy (SEM) observations were conducted at low magnification to record the position of the nanowires. The samples were spin coated with an electron beam sensitive resist and then baked at 180 °C. After this process, the sample was exposed to an electron beam and soaked in a developer in order to create the desired pattern. The chip was next covered with Ti (20 nm) using a DC sputtering technique, respectively. Finally, a lift-off process was used to remove undesirable Ti.

## Additional Information

**How to cite this article**: Park, D. *et al.* Thermal and Electrical Conduction of Single-crystal Bi_2_Te_3_ Nanostructures grown using a one step process. *Sci. Rep.* **6**, 19132; doi: 10.1038/srep19132 (2016).

## Supplementary Material

Supplementary Dataset1

## References

[b1] Lan Y. *et al.* Structure Study of Bulk Nanograined Thermoelectric Bismuth Antimony Telluride. Nano Letters 9, 1419–1422 (2009). 19243189 10.1021/nl803235n

[b2] Dresselhaus M. S. *et al.* New Directions for Low-Dimensional Thermoelectric Materials. Advanced Materials 19, 1043–1053 (2007).

[b3] Kittel C. Introduction to Solid State Physics. 8 edn, (John Wiley & Sons, Inc., 2004).

[b4] Banik A., Shenoy U. S., Anand S., Waghmare U. V. & Biswas K. Mg Alloying in SnTe Facilitates Valence Band Convergence and Optimizes Thermoelectric Properties. Chemistry of Materials 27, 581–587 (2015).

[b5] Biswas K. *et al.* High-performance bulk thermoelectrics with all-scale hierarchical architectures. Nature 489, 414–418 (2012). 10.1038/nature11439 22996556

[b6] Biswas K. *et al.* Strained endotaxial nanostructures with high thermoelectric figure of merit. Nat Chem 3, 160–166 (2011). 21258390 10.1038/nchem.955

[b7] Guin S. N., Chatterjee A., Negi D. S., Datta R. & Biswas K. High thermoelectric performance in tellurium free p-type AgSbSe2. Energy & Environmental Science 6, 2603–2608 (2013).

[b8] Uher C. In Semiconductors and Semimetals Vol. Volume 69 (ed Tritt Terry M.) 139–253 (Elsevier, 2001).

[b9] Yu J.-K., Mitrovic S., Tham D., Varghese J. & Heath J. R. Reduction of thermal conductivity in phononic nanomesh structures. Nat Nano 5, 718–721 (2010). 10.1038/nnano.2010.149 20657598

[b10] Boukai A. I. *et al.* Silicon nanowires as efficient thermoelectric materials. Nature 451, 168–171 (2008). 18185583 10.1038/nature06458

[b11] Cornelius T. W. *et al.* Nanopores in track-etched polymer membranes characterized by small-angle x-ray scattering. Nanotechnology 21, 155702 (2010). 20332555 10.1088/0957-4484/21/15/155702

[b12] Holtzman A., Shapira E. & Selzer Y. Bismuth nanowires with very low lattice thermal conductivity as revealed by the 3ω method. Nanotechnology 23, 495711 (2012). 23154308 10.1088/0957-4484/23/49/495711

[b13] Soini M. *et al.* Thermal conductivity of GaAs nanowires studied by micro-Raman spectroscopy combined with laser heating. 97, 263107 (2010).

[b14] Balandin A. A. *et al.* Superior Thermal Conductivity of Single-Layer Graphene. Nano Letters 8, 902–907 (2008). 18284217 10.1021/nl0731872

[b15] Westover T. *et al.* Photoluminescence, Thermal Transport, and Breakdown in Joule-Heated GaN Nanowires. Nano Letters 9, 257–263 (2008). 19090697 10.1021/nl802840w

[b16] Zhang G., Liu C. & Fan S. Directly measuring of thermal pulse transfer in one-dimensional highly aligned carbon nanotubes. Sci Rep 3, 2549 (2013). 23989589 10.1038/srep02549 PMC3757357

[b17] Yan R. *et al.* Thermal Conductivity of Monolayer Molybdenum Disulfide Obtained from Temperature-Dependent Raman Spectroscopy. ACS Nano 8, 986–993 (2013). 24377295 10.1021/nn405826k

[b18] Wei Q. *et al.* The synthesis of Bi_2_Te_3_ nanobelts by vapor–liquid–solid method and their electrical transport properties. Journal of Materials Science 46, 2267–2272 (2011).

[b19] Zhang G. *et al.* Rational Synthesis of Ultrathin n-Type Bi_2_Te_3_ Nanowires with Enhanced Thermoelectric Properties. Nano Letters 12, 56–60 (2011). 22111899 10.1021/nl202935k

[b20] Bell L. E. Cooling, Heating, Generating Power, and Recovering Waste Heat with Thermoelectric Systems. Science 321, 1457–1461 (2008). 18787160 10.1126/science.1158899

[b21] Kim S. H. & Park B. K. Solvothermal synthesis of Bi_2_Te_3_ nanotubes by the interdiffusion of Bi and Te metals. Materials Letters 64, 938–941 (2010).

[b22] Purkayastha A. *et al.* Surfactant-Directed Synthesis of Branched Bismuth Telluride/Sulfide Core/Shell Nanorods. Advanced Materials 20, 2679–2683 (2008). 25213889 10.1002/adma.200702572

[b23] Wang G., Lok S. K., Wong G. K. L. & Sou I. K. Molecular beam epitaxy-grown Bi4Te3 nanowires. Applied Physics Letters 95, 263102 (2009).

[b24] Hamdou B. *et al.* Thermoelectric Characterization of Bismuth Telluride Nanowires, Synthesized Via Catalytic Growth and Post-Annealing. Advanced Materials 25, 239–244 (2013). 23124978 10.1002/adma.201202474

[b25] Teweldebrhan D., Goyal V. & Balandin A. A. Exfoliation and Characterization of Bismuth Telluride Atomic Quintuples and Quasi-Two-Dimensional Crystals. Nano Letters 10, 1209–1218 (2010). 20205455 10.1021/nl903590b

[b26] Kresse G. & Joubert D. From ultrasoft pseudopotentials to the projector augmented-wave method. Physical Review B 59, 1758–1775 (1999).

[b27] Perdew J., Burke K. & Ernzerhof M. Generalized Gradient Approximation Made Simple. Physical Review Letters 77, 3865–3868 (1996). 10062328 10.1103/PhysRevLett.77.3865

[b28] Chen L., Zhao Q. & Ruan X. Facile synthesis of ultra-small Bi_2_Te_3_ nanoparticles, nanorods and nanoplates and their morphology-dependent Raman spectroscopy. Materials Letters 82, 112–115 (2012).

[b29] Burke H. & Herman I. Temperature dependence of Raman scattering in Ge_1-x_ Si_x_ alloys. Physical Review B 48, 15016–15024 (1993). 10.1103/physrevb.48.15016 10008032

[b30] Liu J. H., Wang H. D., Ma W. G., Zhang X. & Song Y. Simultaneous measurement of thermal conductivity and thermal contact resistance of individual carbon fibers using Raman spectroscopy. Review of Scientific Instruments 84, 044901 (2013). 23635221 10.1063/1.4801495

[b31] Hsu I.-K. *et al.* Optical measurement of thermal transport in suspended carbon nanotubes. Applied Physics Letters 92, 63119 (2008).

[b32] Muñoz Rojo M. *et al.* Fabrication of Bi_2_Te_3_ nanowire arrays and thermal conductivity measurement by 3ω-scanning thermal microscopy. Journal of Applied Physics 113, 054308 (2013).

[b33] Zhang P. *et al.* Electronic transport in nanometre-scale silicon-on-insulator membranes. Nature 439, 703–706 (2006). 16467833 10.1038/nature04501

[b34] Ham J. *et al.* Direct Growth of Compound Semiconductor Nanowires by On-Film Formation of Nanowires: Bismuth Telluride. Nano Letters 9, 2867–2872 (2009). 19588906 10.1021/nl9010518

[b35] Jones P. *et al.* In *Thermoelectrics, 2006*. Electrical Contact Resistance of Bismuth Telluride Nanowires *ICT ‘06. 25th International Conference on.* 693-696.

[b36] Rowe D. M. Thermoelectrics Handbook: Macro to Nano. (CRC Press, 2005).

[b37] Das V. & Soundararajan N. Size and temperature effects on the thermoelectric power and electrical resistivity of bismuth telluride thin films. Physical Review B 37, 4552–4559 (1988). 10.1103/physrevb.37.4552 9945113

[b38] Hsiung T.-C., Mou C.-Y., Lee T.-K. & Chen Y.-Y. Surface-dominated transport and enhanced thermoelectric figure of merit in topological insulator Bi1.5Sb0.5Te1.7Se1.3. Nanoscale 7, 518–523 (2015). 25409984 10.1039/c4nr05376a

[b39] Dayeh S. A., Soci C., Yu P. K. L., Yu E. T. & Wang D. Influence of surface states on the extraction of transport parameters from InAs nanowire field effect transistors. Applied Physics Letters 90, 162112 (2007).

[b40] Dayeh S. A., Soci C., Yu P. K. L., Yu E. T. & Wang D. Transport properties of InAs nanowire field effect transistors: The effects of surface states. Journal of Vacuum Science &amp; Technology B 25, 1432–1436 (2007).

[b41] Lüth H. Solid Surfaces, Interfaces and Thin Films. (Springer Berlin Heidelberg, 2010).

[b42] Kibria M. G. *et al.* Tuning the surface Fermi level on p-type gallium nitride nanowires for efficient overall water splitting. Nat Commun 5, 10.1038/ncomms4825 (2014). 24781276

