# Supplementary material for: Thermal and Electrical Conduction of Single-crystal Bi2Te3 Nanostructures grown using a one step process
Source: Sci Rep. 2016 Jan 11;6:19132. doi: 10.1038/srep19132 (PMC4707524; doi:10.1038/srep19132)
Supplement: Supplementary Dataset1 [file srep19132-s1.doc]

**Thermal and Electrical Conduction of Single-crystal Bi_2_Te_3_ Nanostructures grown using a one step process**

Dambi Park^1^, Sungjin Park^1^, Kwangsik Jeong^1^, Hong-Sik Jeong^2^, Jea Yong Song^3^, and Mann–Ho Cho^1*^

^1^ *Institute of Physics and Applied Physics, Yonsei University, Seoul, 120-749 Korea*

*^2^School of Integrated technology, Yonsei University, Incheon, 406-840 Korea*

*^3^Korea Research Institute of Standards and Science, Daejeon 305-340*

**Supplementary information**

**Supplementary Fig. S1.** The power-dependent Raman spectra of Bi_2_Te_3_ (a) nanowire and (b) nanoribbon.

**Supplementary Fig. S2.** The diameter distributions of Bi2Te3 (a) nanowires and (b) nanoribbons. The diameters of Bi2Te3 nanowires range from 50 to 140nm and the width of Bi2Te3 nanoribbons range from 150 to 220 nm.

**Supplementary Fig. S3.** The n and k vs energy in rhombohedral Bi2Te3 single crystal were obtained using DFT method.
